# Adaptation planning in the context of a weakening and possibly collapsing Atlantic Meridional Overturning Circulation (AMOC)

**DOI:** 10.1007/s10113-025-02434-5

**Published:** 2025-07-02

**Authors:** Robbert Biesbroek, Marjolijn Haasnoot, Katharine J. Mach, Arthur C. Petersen

**Affiliations:** 1 Public Administration and Policy Group, Wageningen University and Research, Hollandseweg 1, 6700 KN Wageningen, The Netherlands; 2 Department of Climate Adaptation and Disaster Risk Management, Deltares, Delft, The Netherlands; 3 Department of Geosciences, Utrecht University, Utrecht, The Netherlands; 4 Department of Environmental Science and Policy, Rosenstiel School of Marine, Atmospheric, and Earth Science, University of Miami, Miami, FL USA; 5 Leonard and Jayne Abess Center for Ecosystem Science and Policy, University of Miami, Coral Gables, FL USA; 6 Department of Science, Technology, Engineering and Public Policy, University College London, London, UK

**Keywords:** AMOC, Adaptation, Tipping point, Pathways

## Abstract

Climate scientists have raised concerns about the weakening of the Atlantic Meridional Overturning Circulation (AMOC) or even its potential collapse in the future. Their messages should not hinder urgent adaptation to climate risks; rather, they underscore the growing need for adaptive planning across a range of possible futures, including high-impact, low-likelihood AMOC scenarios. There are five ways to consider the consequences of AMOC weakening or collapse in adaptation planning: (1) broaden the set of future adaptation scenarios considered; (2) develop adaptation pathways beyond the most likely range of possible outcomes; (3) create robustness and redundancy in adaptation portfolios; (4) expand the solution space, attuned to path dependencies and their implications; and (5) monitor emerging, weak signals of AMOC changes to inform adaptation planning. We argue that closer collaboration between climate scientists and the adaptation planning community is needed to generate timely, policy-relevant insights that can guide proactive and effective adaptation action.

## Introduction

The Atlantic Meridional Overturning Circulation (AMOC) is an important potential climate tipping point (Armstrong McKay et al. 2022). Recently, some studies have suggested that AMOC is showing signs of slowing down, potentially surpassing key thresholds within this century (Ditlevsen and Ditlevsen 2023; Rahmstorf 2024; van Westen et al. 2024). The impacts of such a tipping event are expected to be extremely disruptive to people and ecosystems across the globe and would be particularly consequential in North America and Europe; projections suggest a strong and rapid cooling in Europe that will happen much quicker than the warming seen to date, as well as significant increases in the rate of sea level rise (e.g. an additional 10–25 cm by the end of the century along the US coast) (Liu et al. 2020; Rahmstorf 2024; van Westen et al. 2024). Precipitation patterns could shift, making large parts of Europe dryer than previously projected and other extreme events, including extreme storms, more severe and likely to occur. Some studies note that adaptation responses will face challenges and limits under the rate and severity of impacts resulting from strong AMOC weakening or collapse (van Westen et al. 2024).

Although there remain deep uncertainties in the scientific debates about AMOC, the large-scale potential impacts raise questions about what this means for current and future adaptation action to climate risks. We point to five areas where strong weakening of AMOC or even collapse can be considered in adaptation planning and decision-making.

## Broaden the set of futures for adaptation action

Planning for deeply uncertain and high-impact events requires expanding the range of future scenarios considered and then stress-testing adaptation strategies. AMOC dynamics, including some weakening, have been included already in several CMIP6 Earth System Models, particularly those that model ocean–atmosphere interactions. However, climate impact and risk studies have infrequently examined strong weakening or potential collapse. Yet climate tipping points are considered ‘too risky to bet against’ (Lenton et al. 2019) because of the substantial potential impacts, costs and time needed to take action. Better insights into the magnitude and rate of the impacts of strong AMOC weakening or collapse, and how they might affect people and ecosystems across regions, will be important. This is particularly important in early planning stages, in order to assess the potential relevance of AMOC for long-term adaptation planning and decision-making, for example the time frames that would be necessary to adjust and implement adaptation plans.

Future climate impact studies could also more explicitly explore the potential resulting climatic conditions that fundamentally differ from trends currently considered in adaptation planning (e.g. the substantial possible cooling that could occur), along with impacts beyond the ‘likely’ ranges normally considered (e.g. for sea level rise and droughts). With such robust climate impact studies currently lacking (Lenton and Ciscar 2013), exploring AMOC-induced changes in more qualitative ways still offers possibilities to explore deeply uncertain futures. And although the consequences of such tipping events may not be felt until the end of the century, changes in AMOC are particularly relevant for response measures with a long lifetime and significant societal impact, such as critical infrastructure like seawalls, harbours and energy networks, as well as for urban expansion and adjustments to the existing built environment.

## Create robustness, flexibility and redundancy in the adaptation portfolio

Many of today’s adaptation actions already help address some of the risks associated with a strong weakening or collapse of AMOC, such as increased drought, accelerated sea level rise and more frequent weather extremes. For example, urban greening, including planting trees, expanding parks and integrating green roofs and walls, can moderate urban microclimates, helping cities cope with both hotter *and* colder conditions. However, if AMOC weakens significantly, some of the climate risks previously anticipated for the distant future may materialize much earlier, accelerating the rate at which adaptation limits are reached.

Robust adaptation portfolios are thus essential to navigate such uncertainty. This means adopting a diverse set of adaptation options that ensures resilience across different scenarios and avoids overreliance on a single approach. Many existing adaptation actions will still provide benefits, but they may need to be scaled up, adjusted, or complemented with new strategies as clearer signals of AMOC weakening emerge. This could also require novel transformational adaptation options that have not yet been explored or widely implemented.

At the same time, adaptation portfolios need to be flexible and iteratively adjustable, allowing responses to change if conditions evolve. This means designing sets of measures that can be expanded, modified or phased over time, rather than relying on static solutions that could prove to be costly or difficult to modify. Consider, for example, land reservations to accommodate future expansion for water infrastructure or storage. For situations where this flexibility is more difficult, such as in critical infrastructure, building redundancy is the key. Doing so necessitates adaptation portfolios that can handle risks beyond the most likely ranges of outcomes and that incorporate the ability to ‘fail safely’ where necessary. For instance, certain areas can be designed to temporarily store excess floodwater (e.g. low-lying roads, parking areas or parks), sacrificing their short-term usability to protect homes, hospitals or data centers.

Further enhancing robustness and redundancy of adaptation portfolios inevitably comes with higher socio-economic costs, and some of the adaptation measures may prove to be unnecessary if AMOC remains stable. Such investments are, however, not wasted. Many of the adaptation options strengthen resilience to current climate risks while also offering a broad range of co-benefits; urban greening can improve public health and well-being, raise property values and boost local economies, increase social cohesion by creating shared public spaces, and support biodiversity. Robust and flexible adaptation portfolios can therefore serve as low-regret options, delivering long-term value even if worst-case AMOC scenarios do not materialize.

## Develop adaptation pathways beyond the ‘likely’ range

Planning for future climate risks requires navigating deep uncertainty about how climate and socio-economic systems will evolve over time. Adaptation pathways approaches offer a valuable framework to inform decision-making in the face of such uncertainty, providing flexibility to respond to changing future conditions (Haasnoot et al. 2024). Each adaptation pathway represents a set of adaptation measures and a sequence of decisions which allow for switching between pathways or adjusting strategies as new information becomes available or as conditions evolve. Historically, these approaches have focussed on estimated ‘likely’ climate scenarios. However, given the potential for more drastic climate shifts as result of strong weakening or collapse of AMOC, it is important to expand the scope of these pathways to incorporate more extreme scenarios.

**Fig. 1:**
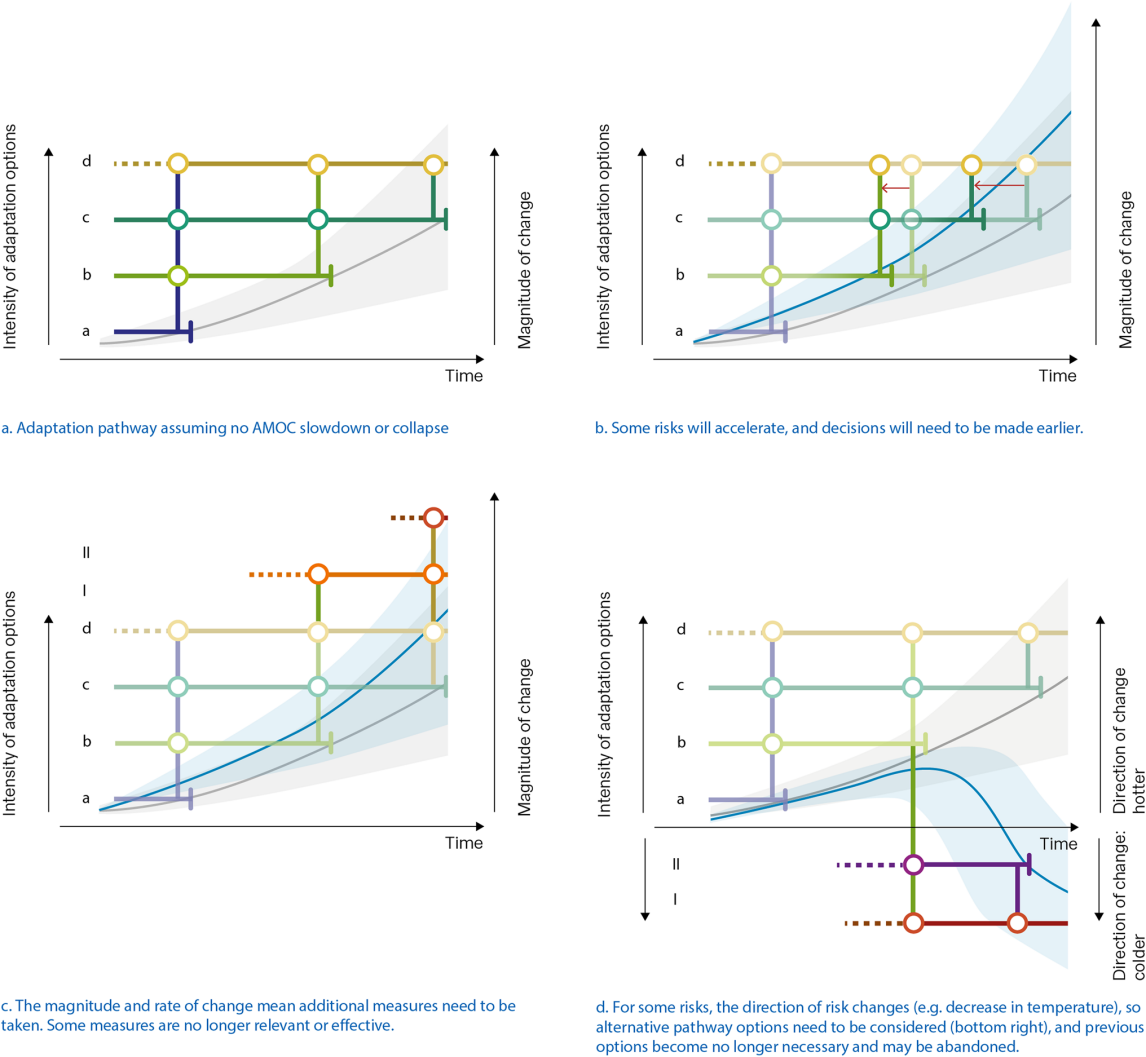
Four examples of how strong weakening or collapse of AMOC impacts adaptation planning. **a** shows an adaptation pathway without significant weakening or collapse of AMOC. **b** shows that AMOC changes may result in some decisions needing to be made earlier. **c** shows that rapid changes may require new or more transformational measures. **d** shows that for some risks the direction of change is shifting, requiring additional measures to be considered

Using these tools, adaptation planning can become more robust, identifying pivotal decision moments in the future where adjustments in approach may be needed. Figure 1a–d illustrate the need for making earlier decisions (Fig. 1b), exploring new sets of more transformative options (Fig. 1c) or considering options that go against the previous trends (Fig. 1d).

These tools help to avoid lock-in to inflexible or maladaptive pathways, and they challenge decision-makers to think more critically about the possible options. This is particularly important as there is a considerable time lag between recognizing the need for new or additional adaptation and the actual implementation of the adaptation measures. Strong weakening or collapse of AMOC is unlikely to occur abruptly but will instead likely unfold over time. Although there will be time to adapt, many of the decisions taken today will have implications beyond mid-century. This is particularly relevant given the time it takes to implement new or additional adaptation measures and the socio-political resistance to rapid, far-reaching adaptation.

A range of tools can be used for designing such types of adaptation pathways. One key approach is combining forecasting and back casting methodology. Forecasting helps to anticipate possible future outcomes, while back casting identifies actionable steps working backwards from future scenarios to the present. These methods have been used in several places already; in the Netherlands, for example, four alternative adaptation futures were developed after an extreme stress test under multiple levels of sea level rise, for which narrative pathways capturing pivotal decisions were identified and later quantified (Haasnoot et al. 2020a, b). Applying these tools to AMOC-related adaptation can provide useful insights, offering a roadmap for decision-making.

## Expand the solution space to ensure accelerated action when needed

Making transformational pathways and a robust adaptation portfolio possible requires an enabling solution space. Some adaptation options or whole adaptation pathways that anticipate AMOC collapse scenarios may fall beyond the current solution space for adaptation, meaning they are not feasible to be implemented at the current time (Haasnoot et al. 2020a). Expanding the solution space is important, but this takes time, is resource intensive, is likely to meet societal and political opposition, and is often beyond the immediate control of government.

Yet several approaches for expanding the solution space have been tried and evaluated, for example building societal awareness of climate tipping points through youth and educational programs and strengthening their engagement; designing policy and legal frameworks that allow for ‘ratcheting up’ and/or that contain ‘review’ or ‘sunset’ clauses based on the latest scientific insights and monitoring (Jordan and Moore 2023); building emergency funding schemes that can be used only when urgent climate action is required; creating durable institutional arrangements by placing them outside the direct influence of line ministries and the political arena; and investing in progressive incremental societal support with increasing returns that over time can lead to substantial change (Levin et al. 2012), for example through investing in policy experiments, research and technological development.

Expanding the solution space is not a panacea, but it is a precondition for engaging with high-impact, low-probability risks such as AMOC collapse, where anticipatory and transformative approaches are essential to avoid maladaptation or delayed crisis responses.

## Monitor and analyze (weak) signals of key indicators

Most AMOC studies recognize the importance of monitoring and analyzing the emerging, weak signals of AMOC changes. Monitoring is most relevant in the context of the physical system, including model-based monitoring and real-time monitoring of indicators that could signal systemic change, such as subtle shifts in sea surface temperature, salinity levels or ice sheet melt (Ditlevsen and Ditlevsen 2023; Sherwood et al. 2024). Given AMOC’s low-likelihood, high-impact consequences, such monitoring of physical systems, despite its ambiguity (Rietkerk et al. 2025), is crucial for adaptation planning.

Interpreting these early signals in a timely, policy-relevant way is as important as collecting the data. Monitoring must be closely linked to decision-making, including clarifying what the observations mean for the scale, magnitude and timing of AMOC-related impacts and future risks. These should be linked to concrete decision points, such as when to trigger contingency planning, adjust infrastructure timelines or revise long-term investment strategies, so that adaptation remains proactive rather than reactive. Recent climate risk assessments, such as the EU Climate Risk Assessment (EUCRA), have occasionally acknowledged tipping points as qualitative ‘wild cards’ (EEA 2024), but these tipping-point assessments have rarely been translated into concrete adaptation planning, whether in National Adaptation Strategies or in the EU’s adaptation strategy and plan.

Furthermore, monitoring should go beyond physical climate indicators to include societal indicators and tipping points, both positive and negative (Otto et al. 2020). This means monitoring shifts in public perception, political will, financial flows and institutional responses that could accelerate or hinder transformational adaptation. Recognizing and anticipating these shifts can help decision-makers expand the solution space and act proactively, rather than waiting for crises to force reactive measures.

## Closing

The science around AMOC is far from settled, but the potential consequences of a strong AMOC slowdown or collapse are too significant to ignore in adaptation planning. If rapid AMOC collapse was to occur, it would almost certainly lead to widespread losses and damages, as adaptation limits, both soft (e.g. financial, institutional) and hard (e.g. biophysical, technological), are likely to be reached in many contexts. As we have highlighted here, there are several approaches in adaptation planning that can help anticipate possible changes, manage deep uncertainties associated with AMOC and prevent it from becoming a ‘predictable surprise’.

Some have raised concerns that emphasizing the potential risks of AMOC collapse—or other climate tipping points—could foster fatalism, fuel misguided fear and anxiety, divert attention from urgent short-term climate actions, or even be misused to justify inaction (Kopp et al. 2025). Clearly there are climate risks that already involve substantial adaptation gaps now and in the future, where current efforts are failing to keep pace with the rate of climate risks (Berrang-Ford et al. 2021). Yet while societal overreaction to AMOC is a risk, societal underreaction is also problematic.

Rather than allowing such deep uncertainty to paralyze adaptation action, we argue it should serve as a call to strengthen and accelerate adaptation globally. AMOC—and other tipping points—should be integrated into planning frameworks that address a wide range of possible futures. Many actions that enhance resilience to AMOC-related consequences, such as flexible infrastructure, diversified resource systems and improved early warning, also strengthen adaptation more broadly. In this sense, preparing for AMOC scenarios need not divert resources from more immediate priorities, but can be approached through low-regret or co-beneficial strategies. The appropriate balance will depend on local context, risk tolerance and available resources, and it will benefit from national and region wide adaptation planning. This also underscores the need for stronger interdisciplinary collaboration where climate and adaptation scientists generate timely, policy-relevant insights that can guide proactive and effective adaptation action.
